# Predicting Real-World Physical Activity in Multiple Sclerosis: An Integrated Approach Using Clinical, Sensor-Based, and Self-Reported Measures

**DOI:** 10.3390/s25061780

**Published:** 2025-03-13

**Authors:** Patrick G. Monaghan, Michael VanNostrand, Taylor N. Takla, Nora E. Fritz

**Affiliations:** 1 Department of Health Care Sciences, Wayne State University, Detroit, MI 48201, USA; patrick.monaghan@wayne.edu (P.G.M.); michael.vannostrand@wayne.edu (M.V.); 2 Neuroimaging and Neurorehabilitation Laboratory, Wayne State University, Detroit, MI 48201, USA; taylortakla@wayne.edu; 3 Translational Neuroscience Program, Wayne State University, Detroit, MI 48201, USA; 4 Department of Neurology, Wayne State University, Detroit, MI 48201, USA

**Keywords:** multiple sclerosis, physical activity, assessment, mobility, real-world function

## Abstract

Multiple sclerosis (MS) is a chronic neurodegenerative disease characterized by mobility impairments that limit physical activity and reduce quality of life. While traditional clinical measures and participant-reported outcomes provide valuable insights, they often fall short of fully capturing the complexities of real-world mobility. This study evaluates the predictive value of combining sensor-derived clinical measures and participant-reported outcomes to better forecast prospective physical activity levels in individuals with MS. Forty-six participants with MS completed surveys assessing fatigue, concern about falling, and perceived walking ability (MSWS-12), alongside sensor-based assessments of gait and balance. Over three months, participants wore Fitbit devices to monitor physical activity, including step counts and total activity levels. Forward stepwise regression revealed that a combined model of participant-reported outcomes and sensor-derived measures explained the most variance in future physical activity, with MSWS-12 and backward walking velocity emerging as key predictors. These findings highlight the importance of integrating subjective and objective measures to provide a more comprehensive understanding of physical activity patterns in MS. This approach supports the development of personalized interventions aimed at improving mobility, increasing physical activity, and enhancing overall quality of life for individuals with MS.

## 1. Introduction

Multiple Sclerosis (MS) is a chronic, neurodegenerative disorder characterized by the demyelination of axons throughout the central nervous system [1]. Due to the underlying pathology of MS, individuals with MS often report significant walking and balance impairments [2,3,4], impacting 70% of the MS community [2]. These impairments can reduce their ability to engage in physical activity, a key factor in maintaining overall quality of life [5,6]. In fact, individuals with MS have lower physical activity levels than the general population, averaging almost 4000 fewer steps per day [7,8,9]. Engaging in physical activity is crucial for individuals with MS, as it provides numerous health benefits, such as reducing fatigue [7], improving walking ability [8], and enhancing overall quality of life [6]. Increasing physical activity may also slow the progression of MS [9]. Therefore, understanding the challenges posed by MS-related mobility impairments is essential for developing effective strategies to promote and sustain physical activity across the MS community.

Traditional clinical assessments have long been the primary tools for predicting physical activity levels in individuals with MS. These methods typically include collecting subjective patient history, evaluating clinical characteristics, and conducting assessments of walking and balance. For example, a lower score on the Expanded Disability Status Scale (EDSS), a clinical scale of disease progression in MS, has been identified as a significant predictor of physical activity in this population [10]. Further clinical assessments of functional mobility, such as balance and walking speed, have also been associated with physical activity [11,12,13]. In addition to these clinical assessments, participant-reported outcomes like fear of falling [14], fatigue [14], and perceived walking capacity [15] have also been shown to impact activity levels. While these traditional assessments provide valuable insights, they often fall short in capturing the full spectrum of real-world physical activity levels experienced by the MS community. Furthermore, self-reported measures, such as the Godin Leisure–Time Exercise Questionnaire [16], are commonly used to assess physical activity in individuals with MS due to their ease of administration; however, they are limited by potential recall bias, and may not accurately reflect real-world activity levels [17]. The limitations of these conventional approaches highlight the need for more accurate and comprehensive predictors of physical activity. Identifying such predictors is crucial for informing targeted interventions that can effectively address the unique mobility challenges faced by individuals with MS, ultimately leading to more personalized and effective treatment strategies.

In recent years, wearable and sensor-based technologies have emerged as valuable tools for assessing functional mobility in individuals with MS [18]. Body-worn inertial measurement units provide portable, validated, reliable, and objective measures of balance and gait that are sensitive to changes in MS [19,20]. For example, sensor-based versions of traditional mobility assessments, such as the instrumented Timed Up and Go (iTUG), sit-to-stand, and gait and balance assessments, offer quantitative insights into motor function [21]. Research has also demonstrated that sensor-derived outcomes, such as backward walking (BW) velocity, can predict prospective physical activity levels in individuals with MS [22]. The addition of clinical sensors to assess gait and balance can greatly improve the prediction of physical activity levels in individuals with MS. Wearable sensors offer more sensitive and objective measures of mobility impairments that might be missed by traditional clinical assessments [23,24]. For instance, recent studies have shown that sensor-based gait and balance evaluations can identify subtle deficits that standard tests, like the Timed 25-Foot Walk, may overlook [23]. Additionally, sensor-derived mobility assessments can address the limitations of self-reported measures, which often fail to capture important details and may compromise sensitivity and accuracy. By combining sensor-based mobility data with patient-reported disability scores, we can enhance the ability to predict physical activity levels in individuals with MS. Therefore, wireless inertial sensor-derived clinical measures not only provide clinically valuable insights into gait and motor function, but also hold promise as a useful tool for predicting future physical activity levels in individuals with MS.

The complex nature of mobility impairments in MS necessitates a multifaceted approach to predicting real-world physical activity. While traditional clinical assessments and self-reported measures provide valuable insights into mobility and physical activity in MS, they have notable limitations. Many existing prediction models rely on subjective reports, which are prone to recall bias and may not accurately reflect real-world activity levels. Similarly, commonly used clinical scales such as the Patient-Determined Disease Steps (PDDS) scale and (EDSS) primarily assess overall disability and walking function in structured settings, but do not fully capture the complexity of daily activity patterns. Rather than replacing these established measures, we propose integrating participant-reported outcomes with objective, sensor-derived clinical mobility measures that may enhance their predictive ability. We propose that the integration of participant-reported outcomes and sensor-derived clinical measures of mobility could significantly enhance our ability to predict prospective physical activity levels in individuals with MS. By adopting this holistic approach, we aim to capture a more comprehensive and nuanced understanding of mobility challenges and physical activity patterns in MS. Notably, more accurate predictions of physical activity could pave the way for highly personalized interventions, tailored to the specific needs of individuals with MS. Such targeted strategies have the potential to more effectively maintain or improve physical activity levels, ultimately leading to better health outcomes and quality of life for the MS community.

Therefore, the aim of this study is to comprehensively assess the predictive value of participant-reported outcomes and sensor-derived clinical measures for prospective physical activity in individuals with MS. By examining these measures both independently and in combination, we seek to develop a more accurate and nuanced approach to predicting real-world physical activity levels. The findings from this study could transform approaches to maintaining and improving physical activity in people with MS, ultimately leading to improved outcomes such as preserving mobility, promoting independence, and enhancing overall health and quality of life.

## 2. Materials and Methods

### 2.1. Participants

All study procedures were approved by the Wayne State Institutional Review Board, and all participants provided informed consent in accordance with the Declaration of Helsinki. The eligibility criteria included being 18 years or older, having a diagnosis of MS based on the McDonald criteria [25], and having a Patient-Determined Disease Steps (PDDS) score ≤ 6, indicating the ability to ambulate with or without an assistive device ≥ 50% of the time [26]. Participants were excluded if they experienced an MS relapse or exacerbation within the past 30 days, used corticosteroids in the past 30 days, had a comorbid neurologic disorder, a condition affecting cognitive or motor function, or were unable to follow study commands.

### 2.2. Participant-Reported Outcomes

Participants completed surveys to provide demographic information and assess disease severity using the PDDS scale. Concern about falling (CAF) was assessed via the Fall Efficacy Scale-International (FES-I), a validated and reliable tool for assessing concern about individuals with MS falling [27,28]. Fatigue impact was assessed using the Modified Fatigue Impact Scale (MFIS), a 21-item self-reported measure [29]. Higher scores on the MFIS indicate a greater impact of fatigue on daily activities. Perceived walking capacity was assessed through the 12-Item Multiple Sclerosis Walking Scale (MSWS-12), a 12-item questionnaire in which participants rate their MS-related mobility limitations over the past 2 weeks using a 5-point scale, ranging from 1 (not at all) to 5 (extremely) [30].

### 2.3. Sensor-Derived Clinical Measures

Participants completed the Timed 25-Foot Walk in both the forward walking (FW) and BW directions. Participants completed two trials in each direction at a self-selected, comfortable walking speed. The FW and BW velocities were then computed and used in the analysis. The T25FW, widely used to assess ambulation disability in individuals with MS, is reliable, valid, and clinically meaningful in MS [22,31].

Participants wore six wireless Opal inertial sensors (128 Hz; APDM Inc., Portland, OR, USA) to measure balance using Mobility Lab’s ISway protocol [32]. They completed two 30 s trials of static balance with eyes closed feet together (ECFT). During this test, participants stood still with arms crossed comfortably on their chests and their eyes closed and feet were placed as close together as possible. This balance task was selected for its complexity, as it manipulates both the base of support and visual input—key factors in balance control [33]. Sway measures were averaged across the two trials. The total sway area was used in the analysis [19]. 

The instrumented push-and-release test assessed reactive balance [34]. Participants stood with feet shoulder-width apart as research staff placed hands on their scapulae and instructed them to lean backward until their body’s midline slightly exceeded their heels in the sagittal plane. After holding this position for 2–5 s, the examiner abruptly removed their hands, prompting participants to regain balance. Reactive balance was quantified using sensor-derived metrics from Opal inertial sensors [35]. Metrics included time to stabilize, defined as the interval between release and the trunk becoming stationary (lumbar sensor acceleration < 7% of gravity and rotational rate < 7°). Outcomes were averaged across three test trials following two familiarization trials. The data were processed using validated MATLAB (version 2023b) algorithms [35,36], previously applied in individuals with MS and other populations, including those with concussion and traumatic brain injury [37,38,39].

### 2.4. Prospective Physical Activity

Participants were provided with a Fitbit Versa 2 smartwatch to passively record physical activity data over a 3-month period using the Fitabase software platform. The Fitbit Versa 2 utilizes a triaxial accelerometer and optical heart rate sensors to estimate movement patterns, including activity intensities (e.g., active minutes at various levels) and metabolic equivalent minutes. These estimates are derived from a combination of basal metabolic rate (adjusted for age, sex, height, and weight), accelerometry data, and heart rate measurements. From the Fitabase (Fitabase, San Diego, CA, USA) platform, we extracted metrics such as sedentary minutes, lightly active minutes, fairly active minutes, very active minutes, and total steps [40,41]. Participants were instructed to wear the Fitbit device as consistently as possible throughout the monitoring period. For physical activity measures, we included both prospective daily step counts and daily total activity levels, as a previous study highlighted their distinction as separate constructs of physical activity and underscored the importance of measuring and reporting domain-specific activity [39]. To ensure valid wear time, adherence was defined as wearing the device for at least 70% of the 3-month period, with a valid day requiring a minimum of 10 h of wear time [42,43,44]. Fitbits have demonstrated reliability and validity in measuring physical activity levels in individuals with MS [45,46]. Studies have shown strong agreement between wrist-worn Fitbit devices and waist-worn ActiGraph devices [47]. Further, the wrist-worn device was selected for its feasibility, comfort, and lower invasiveness during the three-month monitoring period to help ensure compliance.

### 2.5. Statistical Analyses

**Figure 1:**
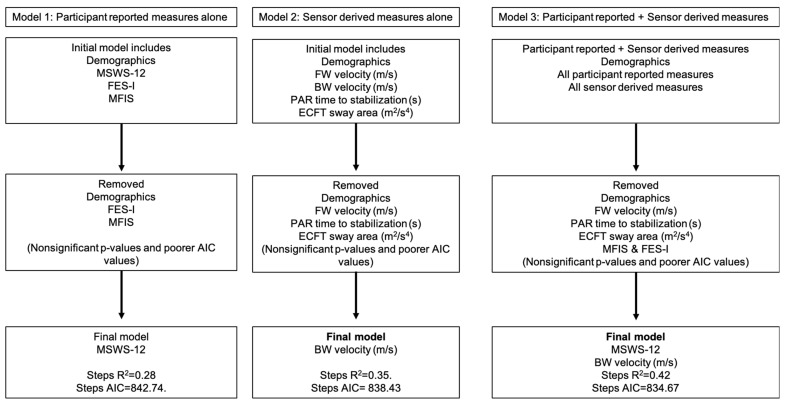
Organizational outline summarizing results from three regression models predicting 3-month total step counts, evaluated using the Akaike Information Criterion (AIC) for goodness of fit. Model 1 included participant-reported outcomes alone, model 2 used sensor-derived clinical measures, and model 3 combined participant-reported outcomes with sensor-derived clinical measures. The best-fitting model (model 3) explained 43% of the variance in 3-month step counts by integrating both participant-reported and clinical measures. Abbreviations: MSWS-12, 12-Item Multiple Sclerosis Walking Scale; BW, backward walking; FW, forward walking; FES-I, Fall Efficacy Scale-International; MFIS, Modified Fatigue Impact Scale; PAR, Push and Release Reactive Balance Assessment; ECFT, eyes closed feet together sway condition; and AIC, Akaike Information Criterion.

All variables are reported as mean ± standard deviation unless otherwise specified. Data normality was assessed by examining skewness and kurtosis values. To evaluate relationships between prospective physical activity, participant-reported outcomes, and sensor-derived measures, Pearson’s correlation coefficients (*r*) or Spearman’s rank correlation coefficients (*ρ*) were calculated, depending on data distribution. Forward stepwise multiple linear regression models were employed to identify significant predictors. Model 1 included patient-reported outcomes, model 2 included sensor-derived measures, and model 3 incorporated both sets of variables (see Figure 1). Model selection was guided by the Akaike Information Criterion (AIC), which accounts for the number of predictors in each model [48]. A lower AIC value indicates a better model fit, allowing for the direct comparison of models with varying numbers of variables. To assess the contribution of individual predictors and the variance explained by each model, standardized beta coefficients (β) and R^2^ values were reported. All regression models were adjusted for age and PDDS. Statistical analyses were performed using SPSS version 29.0 (SPSS Inc., Chicago, IL, USA).

## 3. Results

A convenience sample of 56 individuals with MS was included in the study. A total of 11 individuals did not meet the valid wear time criteria outlined in the methods, so the analyses were conducted in 45 individuals with MS. Table 1 summarizes the demographic and clinical characteristics of the sample. Participants had an average age of 51.16 ± 11.12 years, reported low levels of disability on the PDDS scale (median = 1), and were predominantly female (84%). During the three-month monitoring period, device wear-time compliance was high, with participants wearing the device for an average of 1299 ± 109 min per day (maximum minutes per day = 1440). Participants took an average of 5947 ± 3079 steps per day, reflecting low levels of physical activity that do not meet step count recommendations of 7500 daily steps [49,50].

### 3.1. 3-Month Total Step Count

Regarding 3-month steps per day, significant negative correlations were observed with PDDS (*ρ* = −0.40, *p* < 0.01), MSWS-12 (*ρ* = −0.50, *p* < 0.01), and FES-I (*ρ* = −0.44, *p* < 0.01). These results indicate that higher step counts are associated with lower disease severity (PDDS), better self-reported walking abilities (MSWS-12), and lower concern about falling (FES-I). Significant positive correlations were observed for walking velocity in both the forward (*r* = 0.45, *p* < 0.01) and backward (*r* = 0.57, *p* < 0.01) directions, indicating that higher walking speeds are associated with increased step counts (Table 2).

The results of the forward stepwise linear regressions examining 3-month average daily steps are presented in Table 3 and Figure 1. Model 1 (participant-reported outcomes) was significant (*F*(3,41) = 5.29, *p* < 0.01), and MSWS-12 (*β* = −0.68, T = −2.65, *p* < 0.01) was the only significant predictor remaining after correcting for age and PDDS, with the final model explaining 28% of the variance in the prospective 3-month steps per day (*R*^2^ = 0.28). Model 2 (sensor-derived measures) was also significant (*F*(3,41) = 7.20, *p* < 0.01), and BW velocity was the only significant predictor for the prospective 3-month daily steps (*β* = 0.57, T = 3.44, *p* < 0.01; *R*^2^ = 0.36). Model 3, including both participant-reported and sensor-derived measures, was significant (*F*(4,40) = 7.35, *p* < 0.01), with both MSWS-12 and BW velocity significantly contributing to the model. Specifically, for 3-month steps per day, the inclusion of both variable types explained more variance, as indicated by R^2^ = 0.42.

### 3.2. 3-Month Total Activity 

Regarding 3-month total physical activity, significant negative correlations were observed with PDDS (*ρ* = −0.49, *p* < 0.01), MSWS-12 (*ρ* = −0.59, *p* < 0.01), and FES-I (*ρ* = −0.51, *p* < 0.01). Positive correlations were also seen with forward walking velocity (*r* = 0.50, *p* < 0.01) and backward walking velocity (*r* = 0.54, *p* < 0.01) (Table 2).

**Figure 2:**
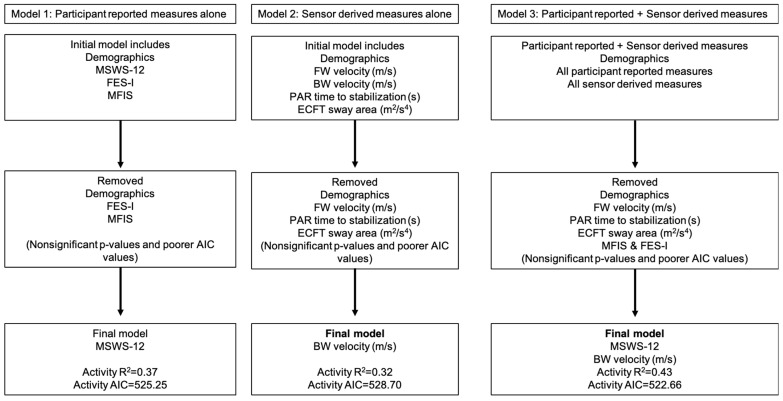
Organizational outline summarizing results from three regression models predicting 3-month total physical activity, evaluated using the Akaike Information Criterion (AIC) for goodness of fit. Model 1 included participant-reported outcomes alone, model 2 used sensor-derived clinical measures, and model 3 combined participant-reported outcomes with sensor-derived clinical measures. The best-fitting model (model 3) explained 43% of the variance in 3-month total physical activity by integrating both participant-reported and clinical measures. Abbreviations: MSWS-12, 12-Item Multiple Sclerosis Walking Scale; BW, backward walking; FW, forward walking; FES-I, Fall Efficacy Scale-International; MFIS, Modified Fatigue Impact Scale; PAR, Push and Release Reactive Balance Assessment; ECFT, eyes closed feet together sway condition; and AIC, Akaike Information Criterion.

The results of the forward stepwise linear regressions examining 3-month total physical activity are presented in Table 4 and Figure 2. Model 1 (participant-reported outcomes) was significant (*F*(3,41) = 7.98, *p* < 0.01), and MSWS-12 (β = −0.74, T = −3.08, *p* < 0.01) was the only participant-reported outcome contributing to the model, accounting for 38% of the variance in 3-month total activity (*R*^2^ = 0.38). Model 2 (sensor-derived measures) was also significant (*F*(3,41) = 6.39, *p* < 0.01), and BW velocity was the only significant predictor of 3-month daily total activity (*β* = 0.41, T = 2.40, *p* = 0.02; *R*^2^ = 0.32). Model 3, which included both participant-reported and sensor-derived measures, was also significant (*F*(4,40) = 7.54, *p* < 0.01), and MSWS-12 and BW velocity significantly contributed to the model. Specifically, for the 3-month daily total activity, the inclusion of both variable types explained more variance, as indicated by *R*^2^ = 0.43 for both outcomes.

## 4. Discussion

This study aimed to comprehensively assess the predictive value of participant-reported outcomes and sensor-derived clinical measures for prospective physical activity in individuals with MS. We found that a combined model, incorporating both participant-reported outcomes and sensor-derived clinical measures, explained the greatest variance in prospective physical activity, including total activity levels and step counts. Notably, significant predictors in the final model included the MSWS-12, a measure of perceived walking ability, and BW velocity, an objective sensor-derived functional assessment. These findings underscore the importance of integrating subjective and objective measures to more effectively predict future physical activity. This is impactful, as many individuals with MS do not meet physical activity guidelines, despite their benefits, including reduced fatigue, improved mobility, and enhanced quality of life. Identifying sensitive predictors supports tailored interventions to enhance independence and well-being, enabling proactive strategies to preserve mobility and quality of life.

Our findings underscore the importance of a comprehensive, multifaceted approach to predicting physical activity in individuals with MS. By demonstrating the complementary value of sensor-derived clinical measures and participant-reported outcomes, our study builds on and extends previous research, which has often focused on these domains in isolation. For instance, a cluster of participant-reported outcomes such as perceived walking capacity, pain and depression, and fatigue were associated with physical activity [51,52]. Prior work has also shown that the frequency and severity of participant-reported MS symptoms [53] and fear of falling [54] also influence physical activity levels in MS, emphasizing the critical role of subjective experiences in understanding engagement levels. Similarly, clinical mobility measures have also been shown to be associated with physical activity [13,22], showing that objective assessments like walking speed and balance are strongly associated with activity levels. Our findings uniquely contribute to this body of work by integrating these two perspectives, demonstrating that their combined predictive value exceeds what either can achieve alone. Sensor-derived measures offer the precise, objective quantification of mobility function, such as backward walking velocity, which is particularly sensitive to subtle impairments and predicting prospective activity. In contrast, participant-reported outcomes capture subjective factors—like confidence in walking ability and perceived limitations—that directly influence an individual’s willingness and ability to engage in physical activity. This integrated approach reflects the growing consensus in MS research that mobility impairments are inherently multidimensional and cannot be fully understood through single-metric assessments [39]. By aligning objective and subjective measures, our study provides a more nuanced framework for predicting physical activity, supporting the development of targeted, participant-centered interventions. These results further underscore the need for the continued exploration of hybrid models to address the complex interplay of factors influencing physical activity in MS. 

Previous studies have shown that older adults and individuals with MS or Parkinson’s disease often experience a significant mismatch between their perceived physical limitations and actual functional capacity, which can profoundly impact their engagement in physical activity [55,56]. For example, Gunn et al. reported that 50% of individuals with MS displayed a notable disparity between their perceived and physiological fall risk, highlighting the need to consider both dimensions when evaluating fall risk in this population [55]. This discordance may be particularly pronounced in neurological conditions, where factors such as cognitive impairments, fatigue, and psychological barriers can heavily influence willingness to engage in physical activity, regardless of physiological ability. By integrating objective sensor-derived measurements with participant-reported outcomes, our study provides a holistic framework that captures both the physiological and psychological aspects of mobility. This comprehensive approach not only improves the accuracy of physical activity predictions, but also sheds light on the intricate relationship between perceived and actual functions.

Interestingly, the only significant predictor from the participant-reported outcomes model was the MSWS-12, a measure of perceived walking capacity. Increases in perceived walking capacity were associated with more total activity and more steps. This outcome corroborates previous work that has also found significant associations between MSWS-12 and physical activity in MS [15,57]. This supports prior work that has established an individual’s perception of their capacity as well as the MS-related impairment and disability as being dimensions of physical activity barriers in MS [58], highlighting the utility of such outcomes in predicting those at risk of physical inactivity. It is important to consider that other assessments included within this model, such as the MFIS, were not significant predictors. 

While self-reported fatigue is frequently considered a barrier to engaging in physical activity for individuals with MS [14,59], findings on this relationship are inconsistent. Some studies have found a link between higher fatigue and lower physical activity [60,61], while others report minimal or no association [62,63,64]. Inconsistencies between self-reported fatigue and physical activity levels in MS may arise from several factors. Inconsistencies in the definitions of fatigue and the wide range of fatigue scales used across studies can lead to differing results [65,66]. Notably, our cohort had a relatively low average MFIS score (mean = 33, SD = 18), while previous studies have used a cutoff of 38 to distinguish fatigued from non-fatigued individuals [29,67]. This suggests that our sample may not fully represent the broader MS population, highlighting the need for future studies to include a wider range of fatigue levels in individuals with MS. Additionally, participant characteristics, including age, disability levels, psychological factors such as anxiety and depression, and differences in MS subtypes, may influence the relationship between fatigue and physical activity. 

While previous studies have found a significant correlation between higher FES-I scores and lower physical activity levels in people with MS [54], FES-I was not a significant predictor in our model. This discrepancy may be context-dependent, influenced by differences in how physical activity is measured. Prior studies often relied on self-reported activity, which may lack accuracy or sensitivity and differ in its correlation with objective measures [68]. In contrast, our study used accelerometers to capture activity, including total steps and the sum of light, moderate, and vigorous activity minutes. These objective measures may better reflect actual activity levels, but could explain the lack of association with FES-I in our findings. Moreover, a one-time assessment of CAF using the FES-I may not fully capture fluctuations in concern of falling over time. Future studies should consider ongoing assessments of CAF alongside week-to-week physical activity tracking to better understand their dynamic relationship. 

BW velocity emerged as the only significant sensor-derived clinical predictor of prospective steps and activity minutes, outperforming traditional assessments such as forward walking and standing balance. This finding highlights the potential utility of BW as a clinical assessment with predictive validity for future activity levels. Consistent with prior research showing that better BW performance correlates with higher prospective vigorous activity in individuals with MS [22], this outcome underscores the unique demands of backward walking. It challenges the postural control system [69], engages multi-domain cognitive functioning [70,71], relies heavily on proprioceptive sensory input [72,73], and effectively differentiates MS fallers from non-fallers [74]. These characteristics may make BW a more sensitive predictor of prospective physical activity, reflecting the dynamic and complex environments where activity occurs. 

This study has several limitations that should be considered. First, the majority of participants had relapsing–remitting MS (Table 1), the most common subtype of the disease [75]. While this enhances the relevance of our findings to this population, it may limit generalizability to individuals with other MS subtypes, such as secondary progressive or primary progressive MS. Second, despite participants being instructed to wear the Fitbit device as much as possible, there was variability in adherence to wear time, which may have influenced physical activity outcomes. However, as shown in Table 1, the overall adherence and wear time rate were reasonably compliant across all participants. Third, although our final models did not include psychological factors such as anxiety and depression [76], these variables may also influence the relationship between clinical measures and physical activity. For example, anxiety and depression are common in MS, with prevalence rates significantly higher than in the general population. Depression affects 27–54% of MS patients, while anxiety disorders impact up to 45% [76,77]. These conditions reduce quality of life, correlate with greater disability and fatigue, and discourage physical activity [78,79], meaning that individuals with MS experiencing higher depression and anxiety are less likely to adhere to the exercise regimens essential for managing their condition [78]. Therefore, future work should incorporate these factors to provide a more comprehensive understanding of the determinants of physical activity in MS.

## 5. Conclusions

This study highlights the novel integration of participant-reported outcomes and sensor-derived clinical measures to predict prospective physical activity in individuals with MS. Our findings reveal that combining these domains provides superior predictive power compared to using either in isolation, addressing a critical gap in the understanding of factors influencing real-world activity levels. Specifically, BW velocity and perceived walking capacity emerged as significant predictors, emphasizing the importance of both objective functional performance and subjective perceptions of mobility. By demonstrating the complementary strengths of these measures, our work advances the field toward more comprehensive and accurate prediction models. This approach has the potential to inform targeted, individualized interventions aimed at preserving mobility and promoting engagement in physical activity, thereby improving health outcomes and quality of life for individuals with MS.

## Figures and Tables

**Table 1:** Demographics.

Descriptive Statistics	N = 45
Age (years)	51.16 ± 11.12
Sex (*n*, % female)	38, 84%
Race (*n*)	White: 23 African American/Black: 21 Native American: 2 Pacific Islander: 1 Hispanic/Chicano: 1
PDDS [median (range)]	1 (0–6)
MS Subtype (*n*, %)	RRMS: 42, 93.33% SPMS: 2, 4.44% PPMS: 1, 2.22%
Participant-Reported Outcomes	
MSWS-12	35.69 ± 30.15
FES-I	27.20 ± 9.30
MFIS	32.71 ± 17.79
Sensor-Derived Clinical Outcomes	
BW velocity (m/s)	0.65 ± 0.33
FW velocity (m/s)	0.99 ± 0.37
PAR time to stabilization (s)	1.47 ± 0.64
ECFT sway area (m^2^/s^4^)	0.38 ± 0.46
3-Month Prospective Physical Activity	
Average daily physical activity in minutes	252 ± 93
Average daily step count	5947 ± 3073
Average daily wear time (minutes) (maximum = 1440 min)	1299 ± 109
Average daily percentage wear time	90.25 ± 7.62%

Note. Three individuals reported two racial groups. PDDS: Patient-Determined Disease Steps; MSWS-12: 12-Item Multiple Sclerosis Walking Scale; BW: backward walking; FW: forward walking; FES-I: Fall Efficacy Scale-International; MFIS: Modified Fatigue Impact Scale; PAR: Push and Release Reactive Balance Assessment; and ECFT: eyes closed feet together sway condition.

**Table 2:** Correlation matrix.

	3-Month Step Count	3-Month Total Activity	Age	PDDS	MSWS-12	MFIS	FES-I	FW Velocity	BW Velocity	PAR Time to Stabilization	ECFT Sway Area
3-Month daily step count	-	0.75 **	−0.26	−0.40 **^a^	−0.50 **^a^	−0.27	−0.44 **^a^	0.45 **	0.57 **	−0.27	−0.11 ^a^
3-Month daily total activity		-	−0.11	−0.49 **^a^	−0.59 **^a^	−0.26	−0.51 **^a^	0.50 **	0.54 **	−0.23	−0.01 ^a^
Age			-	0.19 ^a^	0.18 ^a^	−0.05	0.16 ^a^	−0.04	−0.20	0.15	0.14 ^a^
PDDS				-	0.87 **^a^	0.60 **^a^	0.62 **^a^	−0.62 **^a^	−0.67 **^a^	0.30 *^a^	0.54 **^a^
MSWS-12					-	0.68 **^a^	0.63 **^a^	−0.65 **^a^	−0.61 **^a^	0.17 ^a^	0.41 **^a^
MFIS						-	0.55 **^a^	−0.32 *	−0.28 *	0.21	0.40 **^a^
FES-I							-	−0.56 **^a^	−0.57 **^a^	0.32 *^a^	0.36 **^a^
FW velocity								-	0.87 **	−0.29 *	−0.25 ^a^
BW velocity									-	−0.28	−0.46 **^a^
PAR time to stabilization										-	0.27 *^a^
ECFT sway area											-

* Denotes significance *p* < 0.05; ** denotes significance *p* < 0.01; and ^a^ denotes Spearman’s rank correlation. PDDS: Patient-Determined Disease Steps; MSWS-12: 12-Item Multiple Sclerosis Walking Scale; MFIS: Modified Fatigue Impact Scale; FES-I: Falls Efficacy Scale-International; FW: forward walking; BW: backward walking; PAR: Push and Release Reactive Balance Assessment; and ECFT: eyes closed feet together sway condition.

**Table 3:** Linear regression results examining contributors to 3-month daily step count.

3-Month Daily Step Count					
Model 1		B	β	T	*p*-value
	Age	−55.56	−0.20	−1.45	0.15
	PDDS	426.09	0.28	1.08	0.29
	MSWS-12	−65.32	−0.68	−2.65	0.01
	R^2^		0.28		
	AIC		842.74		
	*p*-value		<0.01		
	F-statistic (3,41)		5.29		
Model 2		B	β	T	*p*-value
	Age	−43.08	−0.15	−1.19	0.24
	PDDS	87.73	0.06	0.35	0.73
	BW velocity	5383.30	0.57	3.44	<0.01
	R^2^		0.35		
	AIC		838.43		
	*p*-value		<0.01		
	F-statistic (3,41)		7.20		
Model 3		B	β	T	*p*-value
	Age	−39.56	−0.14	−1.25	0.26
	PDDS	745.32	0.49	2.02	0.05
	MSWS-12	−52.98	0.51	−2.34	0.03
	BW velocity	4777.85	−0.55	3.17	<0.01
	R^2^		0.42		
	AIC		834.67		
	*p*-value		<0.01		
	F-statistic (4,40)		7.35		

PDDS: Patient-Determined Disease Steps; MSWS-12: 12-Item Multiple Sclerosis Walking Scale; BW: backward walking; and AIC: Akaike Information Criterion.

**Table 4:** Linear regression results examining contributors to 3-month daily total activity.

3-Month Daily Activity Count					
Model 1		B	β	T	*p*-value
	Age	−0.22	−0.03	−0.20	0.84
	PDDS	8.05	0.17	0.70	0.49
	MSWS-12	−2.23	−0.74	−3.08	<0.01
	R^2^		0.37		
	AIC		525.25		
	*p*-value		<0.01		
	F-statistic (3,41)		7.98		
Model 2		B	β	T	*p*-value
	Age	−0.05	−0.01	−0.04	0.97
	PDDS	−10.02	−0.21	−1.24	0.22
	BW velocity	120.34	0.41	2.40	0.02
	R^2^		0.32		
	AIC		528.70		
	*p*-value		<0.01		
	F-statistic (3,41)		6.39		
Model 3		B	β	T	*p*-value
	Age	0.08	0.01	0.08	0.94
	PDDS	14.57	0.30	1.26	0.21
	MSWS-12	−1.98	−0.66	−2.80	0.01
	BW velocity	97.70	0.33	2.07	0.05
	R^2^		0.43		
	AIC		522.66		
	*p*-value		<0.01		
	F-statistic (4,40)		7.54		

PDDS: Patient-Determined Disease Steps; MSWS-12: 12-Item Multiple Sclerosis Walking Scale; BW: backward walking; and AIC Akaike Information Criterion.

## Data Availability

The data presented in this study are available upon request from the corresponding author.
